# Acupuncture for patients with mild to moderate Alzheimer’s disease: a randomized controlled trial

**DOI:** 10.1186/s12906-017-2064-x

**Published:** 2017-12-29

**Authors:** Yujie Jia, Xuezhu Zhang, Jianchun Yu, Jingxian Han, Tao Yu, Jiangwei Shi, Lan Zhao, Kun Nie

**Affiliations:** First Teaching Hospital of Tianjin University of Traditional ChineseMedicine, Tianjin, 300193 China

**Keywords:** Acupuncture, Alzheimer’s disease, Randomized controlled trial

## Abstract

**Background:**

Alzheimer’s disease (AD) is the most common cause of dementia. However, none of medical treatment can stop or reverse the underlying neurodegenerative of AD at present. Acupuncture has attracted more and more attention in recent years due to its efficacy and very few side effects. Lately, a systematic review has thought that the evidence on the effectiveness of acupuncture in improving the cognitive function of AD patients was not powerful enough. Therefore, the aim of this study is to explore the efficacy and safety of acupuncture in patients with mild to moderate AD.

**Methods:**

This was a randomized, controlled, parallel-group, exploratory study with 4-week baseline (T0), 12-week treatment phase (T1) and 12-week follow-up period (T2). Patients with mild to moderate AD meeting the included criteria were randomly allocated into either acupuncture or donepezil hydrochloride groups. The acupuncture group(AG) was given acupuncture treatment three times per week and the donepezil hydrochloride group(DG) group was administered donepezil hydrochloride once daily (5 mg/day for the first 4 weeks and 10 mg/day thereafter). Primary efficacy was measured using Alzheimer’s disease Assessment Scale-Cognitive (ADAS-cog) and Clinician’s Interview-Based Impression of Change-Plus (CIBIC-Plus). The second outcomes were measured with 23-Item Alzheimer’s disease Cooperative Study Activities of Daily Living Scales (ADAS-ADL_23_) and Neuropsychiatric Index (NPI).

**Results:**

Of 87 participants enrolled in the study, 79 patients finished their treatment and follow-up processes. The ADAS-cog scores for AG group showed obvious decreases at T2 and ∆(T2-T0)when compared with DG group, and significant between-group differences were detected (all *p* < 0.05). The mean CIBIC-Plus values for the AG group at T1 and T2 were much lower than that for the DG group, and there were significant differences between the two groups (푃<0.05). There were no significant between-group differences in the scores of ADAS-ADL_23_ and NPI during the study period. Treatment discontinuations due to adverse events were 0 (0%) and 4 (9.09%) for the AG and DG groups, respectively.

**Conclusions:**

Acupuncture is safe, well tolerated and effective in improving the cognitive function, global clinical status of AD.

**Trial Registration:**

ChiCTR-IOR-17010465 (Retroactively registered on 18 JAN 2017).

## Background

Alzheimer’s disease (AD) is a slowly progressive neurodegenerative disorder characterized by memory impairment and other cognitive disabilities, personality changes [1].AD is the most common cause of dementia, accounting for 60~70% of all dementia cases [2].Age is the most important risk factor for AD. The prevalence of AD rises exponentially in elderly people from 1% at age of 65 to approximately 40–50% by the age of 95 [3].Moreover, the health care costs for AD are expensive, which have reached $172 billion per year in the United States alone [4].It is estimated that 60% of AD patients live in developing countries, with the proportion being raised to more than 70% by 2040 [5].It can be predicted that AD represents a major public health challenge as a consequence of rapid increase in the aging population worldwide, especially in developing countries.

More and more people pay attention to AD because the decline in memory and other cognitive functions leads to a loss of independence which has a wide-ranging impact on individuals, families and healthcare systems [6].Thus, there is a need for prospective studies to clarify the pathogenesis of this condition and to provide appropriate measures for prevention and treatment of AD. Although AD had already been described about 100 years ago and we have paid enormous research efforts, at present none of medical treatment can stop or reverse the underlying neurodegenerative of AD [7].The medications currently approved by the Food and Drug Administration and European Agency for the Evaluation of Medical Products include cholinesterase-inhibitors(CI) and memantine, which offer modest symptomatic relief [8].A systematic review of the literature has revealed that CI resulted in significantly higher rates of adverse events and discontinuation of treatment [9].At the same time the cost effectiveness of the currently available therapeutic agents for AD has undergone great scrutiny and remains controversial, especially outside the united states of American(USA) [10]. So we should find a treatment that will not only yield maximum benefits for individual patients across multiple domains, including cognition, daily function and behavior, but also provide realistic expectations for patients and caregivers throughout the course of the disease [11].

Acupuncture, as a style of traditional Chinese medicine(TCM), has attract more and more attention in recent years due to its efficacy and very few side effects [12]. However, a new systematic review has thought that the evidence on the efficacy of acupuncture in improving the cognitive function of AD patients was not powerful enough [13]. Another systematic review has also considered that existing evidence didn’t demonstrate the effectiveness of acupuncture for AD [12]. Therefore, the randomized, controlled clinical trial was designed to further determine the effectiveness and safety of acupuncture among patients with mild to moderate AD.

## Methods

### Trial design, setting, randomization and blinding

The study was a randomized, drug-controlled, parallel-group trial to determine the efficacy and safety of acupuncture compared with donepezil in patients with mild to moderate AD from November 2015 to May 2016. All participants came from the older residents with dementia in an epidemiological investigation. The survey was carried out in the community of Simianzhong Street, Heping District, Tianjin City from August to October 2015.The study would last 28 weeks: a 4-week prospective baseline period, 12-week treatment period, and a 12-week follow-up period. The cognitive abilities, activities of daily living and behavioral symptoms of patients would be evaluated before treatment, after 12 weeks, and after 24 weeks using a variety of neuropsychological tests. Overall clinical status was measured at week 16, and 28. After the baseline, all patients were randomly allocated into either AG or DG in a ratio of 1:1 by a computer-generated, randomly location sequence (random list generated with SPSS 13.0) under the help of a professional statistician. The random number table was sealed in a special envelope.

In this study, physicians could not be blinded due to the nature of the intervention. The acupuncturists had to know the group assignment because of manipulation. Data collection was performed by 2 blinded evaluators. Blinding was also maintained for data analysis. Apart from the differences in treatment methods between the two groups, all subjects were treated as equally as possible. During the intervention, acupuncturists and data collection staff would visit patients at different time so that they could not exchange information with each other.

The study was performed according to common guidelines for clinical trials (Declaration of Helsinki, International Conference on Harmonization /WHO Good Clinical Practice standards including certification by an external audit).The trial protocol had been approved by the Research Ethical Committee of First Teaching Hospital of Tianjin University of Traditional Chinese Medicine (TYLL2015[K]003) on July 30, 2015.

### Participants

This study included residents aged 50 to 85 years and main types of surveys were home interview. The screening process consisted of the following three phases: the first phase: personal information of the residents (including age, sex, occupation, weight, marital status, education level, previous medical history, family medical history, living habits, subjective emotion and combined disease) were collected and neuropsychological scales(Mini-mental State Examination, MMSE and Activity of Daily Living scale, ADL) were evaluated. The second phase: the residents whose MMSE and ADL scores were lower than standard were further assessed using 17-item Hamilton depression scale(HAMD) and Hanchinski ischemia scale(HIS). The third phase: physician of neurology made a diagnosis based on medical history and nervous system medical examination.

Diagnostic criteria designed to identify patients with probable AD were developed by the Diagnostic and Statistical Manual of Mental Disorders, Fourth Edition and National Institute of Neurological and Communicative Disorders and Stroke-Alzheimer’s Disease and Related Disorders Association criteria [1]. The patients included in this study were required to have a MMSE score between 10 and 23 [14], a HAMD score<7,a HIS score ≤ 4, and have a reliable caregiver who is able to accompany the participant to all study visits. Individuals were excluded if they had evidence of other diseases which could lead to dementia other than AD; significant unstable psychiatric or neurologic disorders; clinically relevant uncontrolled or unstable gastrointestinal, renal, hepatic, endocrine or cardiovascular disease; clinically significant obstructive pulmonary disease; B12 or folic acid deficiency; alcoholism or drug misuse or current systemic illness influencing cognitive assessment; a history of hematologic and oncologic disorders in the past 2 years. Subjects could not participate if they had received any treatments which probably improve cognitive function within the past 1 month and known sensitivity to acupuncture or Donepezil Hydrochloride. Patients were also excluded from the study if they were incapable of giving written informed consent from a legal representative of the participants.

### Interventions

The treatment strategies for acupuncture was initiated by Professor Jingxian Han during long theoretical study and clinical experience according to the Sanjiao theory of TCM. Then, three experienced acupuncture specialists (zhilongzhang, jianchunyu, yong tang) discussed and finalized the treatment strategies in a consensus process. The acupuncture therapy was performed by nine acupuncturists who are registered Chinese medicine practitioners of the Ministry of Health of the People’s Republic of China and have more than 6 years (median 7.2 years) of clinical experience. The sterile, disposable needles (Huatuo, Suzhou Medical Instruments Factory, China) were used in this study with a diameter of 0.25 mm and a length of 40 mm. These acupoints were employed as basic acupuncture formulas, including RN17(danzhong), RN12(zhongwan), RN6(qihai), ST36(zusanli), SJ5(waiguan) and SP10(xuehai). Moreover, the following acupoints could be selected as auxiliary acupoints according to patient’s symptoms and tongue manifestation,LR3(taichong),GB39(xuanzhong),ST40(fenglong),BL17(geshu),ST44(neiting),ST25(tianshu) andRN4(guan yuan). Except for RN17, RN12, RN6, RN4, the other acupoints were bilateral. Acupuncture prescriptions were individualized to each patient, and different points were used based on the discretion of the acupuncturist.

During acupuncture, patients would have a needling feeling of numbness, tingling, swelling or muscle weakness, which is known as “de qi” and considered as indicators of effective needling. To evoke needle sensation, the needles were inserted obliquely and upward 15 mm into RN17, 15~25 mm perpendicularly into RN12, RN6 and ST36, then rotated at small-amplitude and high-frequency with reinforcing method for 30s. The needles were inserted perpendicularly 15~25 mm into SJ5, then rotated with normal reinforcement and normal reduction method for 30 s. For SP10, the needle was inserted obliquely 15~25 mm into the acupoint, then rotated with big-amplitude and low-frequency reducing method for 30s. The needles would be retained in situ for 30 min. Acupuncture treatment was given three times weekly for 12 weeks. Any additional therapies for AD were not permitted during the entire study period, and only necessary explanations were provided to patients to avoid biased data. All acupuncturists received operation instructions from professor Han, a videotape, and a brochure with detailed information on acupuncture treatment. The patients in the DG group received 5 mg/day of donepezil hydrochloride(Aricept®, Weicai (China) Pharmaceutical Co., Ltd) for the first 4 weeks and 10 mg/day thereafter [15].

### Outcome measures

The primary outcome measures were (1) ADAS-cog, which consists of 11 tasks to assess multiple cognitive domain including memory, language, praxis and orientation. ADAS-cog score ranges from 0 to 70 with higher scores indicating greater impairment. (2)CIBIC-Plus, which mainly assesses treatment effect on overall clinical status of AD patients with scores ranging from 1(marked improved) to 7(markedly worse) [16].

The second outcome measures were (1) ADAS-ADL_23_, which is a test to assess basic and instrumental activities of daily living. The ADAS-ADL_23_ score ranges from 0 to 78, and higher scores indicate lower levels of activities of daily living [17]; (3) NPI, which is a condition-specific measure to assess frequency and severity of behavioral symptoms in patients with dementia. It covers 10 neuropsychic symptoms and 2 autonomic nervous symptoms with total scores ranging from 0 to 144 (higher numbers indicating more behavioral disturbance) [18].

The safety parameters were assessed during each visit, including spontaneously reported adverse events (AEs) or serious AEs (SAEs), vital signs (temperature, heart rate and blood pressure) and physical examination.

### Sample size

According to the results of previous study, an improvement of ADAS-Cog scores for the acupuncture and control groups were 6.99 (standard deviation: 3.23) and 4.02 (standard deviation: 2.11), respectively [19]. Therefore, the number of sample size was calculated using the following formula:

$$ {\mathrm{n}}_1={\mathrm{n}}_2=2{\left[\frac{\left({t}_{\alpha }+{t}_{\beta}\right)s}{\delta}\right]}^2\left(\alpha =0.05,\kern0.5em \beta \kern0.5em =\kern0.5em 0.10\right) $$

As a result, we estimated that 17 patients were required in each group. A 20% drop-out rate was assumed, therefore, 21 patients should be enrolled in each arm.

### Statistical analysis

The results were presented as mean ± SD or frequencies and percentages, according to the type of variables. Demographic data were analyzed using standard statistical tests (e.g., t-test, Fisher’s exact test and binomial test) according to two-tailed *p*-values. For the between-group comparisons at baseline, either the Chi-Square test or one-way covariance was used. Chi square test was applied for count data, and independent samples t-test was used for measurable variables. Efficacy outcome parameters were analyzed on an intent-to-treat basis. The missing data were not disposed. Repeated measures analysis of variance was used to determine whether significant differences exist across time (except CIBIC-Plus).Two-independent-samples t-test was performed when the difference met normal distribution. All AEs were coded using a World Health Organization (WHO)-based dictionary of preferred terms. The numbers (percentages) of patients with at least one AE, at least one SAE, and at least one AE leading to discontinuation were summarized irrespective of relationship to treatment. All safety-related observations were summarized using descriptive statistics. The *P* values lower than 0.05 were considered to be statistically significant. All statistical analysis was performed using SPSS software version 13.0(SPSS Inc., Chicago, USA).

## Results

### Participants

**Fig. 1:**
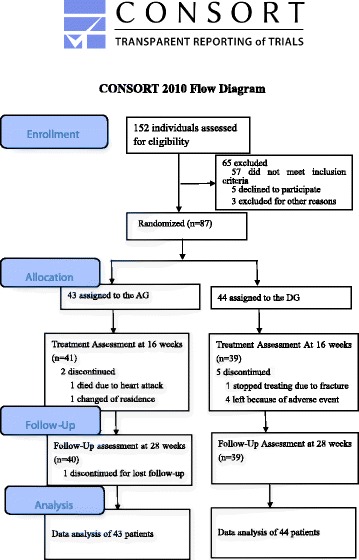
Flowchart of the study design (based on CONSORT 2010 guidelines)

The flow of participants through the trial was shown in Fig. 1. Form November 2015 to May 2016, 152 patients were screened for eligibility. Of these, 57 could not be included because they did not meet all eligibility criteria. A total of 87 patients were enrolled and randomly assigned to either AG (*n* = 43) and DG (*n* = 44) groups. Eight(9.19%) out of 87 patients dropped out during the trial, one patient stopped the treatment due to fracture, one patient died from heart attacks, four patients could not tolerate donepezil for SAEs, one patients withdrew owing to the change of address, and one patient lost at the follow-up. A total of 79 patients (90.80%) completed the entire treatment and follow-up processes, 40 (93.02%) in the AG and 39(88.64%) in the DG.

There were no significant differences in the demographic characteristics (age, sex, education year, marital status, et al) and baseline efficacy parameters (ADAS-cog, ADCS-ADL_23_ and NPI) of the subjects initially enrolled in the study between the two patient cohorts (Table 1).

**Table 1:** Baseline characteristics of study participants

	Acupuncture	Donepezil	total
Gender			
Men n (%)	13(30)	16(36)	29(33)
women n (%)	30(70)	28(64)	58(67)
Age, y, M (SD)	75.11(6.53)	74.50(6.83)	
Education(year)			
0n (%)	6(14)	6(14)	12(14)
1–6 n (%)	6(14)	10(23)	16(18)
7–9 n (%)	22(51)	20(45)	42(48)
≥ 10 n (%)	9(21)	8(18)	17(20)
Severity of dementia			
Mild n (%)	24(56)	23 (52)	47 (54)
Moderate n (%)	19 (44)	21 (48)	40 (46)
times since symptoms first started, M (SD)	4.46 (0.99)	4.42 (1.06)	
Associated diseases n (%)			
Hypertension	19 (44)	17(39)	36 (41)
Diabetes	15(35)	13 (30)	28(32)
Coronary heart disease	12(28)	14 (32)	26 (30)
Insomnia	10 (23)	13 (30)	23(26)
Constipation	12(28)	9 (20)	21 (24)
Benign prostatic hyperplasia	7 (16)	9(20)	16 (18)
Osteoarthritis of the knee	4 (9)	5 (11)	9 (10)
ADAS-cog score, mean (SD)	29.38(9.43)	30.15(9.16)	
ADCS-ADL23 score, mean (SD)	49.44(11.62)	50.13(13.57)	
NPI score, mean (SD)	9.28(2.49)	8.97(2.69)	

### Outcome measures

Mean scores of ADAS-cog, CIBIC-Plus, ADCS-ADL_23_ and NPI at all time points and the change scores between baseline (T0) and week16 (T1), and between T0 and week 28 (T2) for the two groups were shown in Table 2.

**Table 2:** Mean scores at all time points and change scores for ADAS-cog, CIBIC+, ADCS-ADL_23_ and NPI

	Group	T0 baseline	T1 16 weeks	T2 28 weeks	∆ (T1-T0)	∆ (T2-T0)
ADAS-cog	AG	29.38 (9.43)	25.30 (11.33)	26.28 (10.73) ^a^	4.08 (7.73)	3.10 (6.01) ^a^
	DG	30.15 (9.16)	28.18 (9.78)	31.25 (10.71)	1.97 (2.08)	−1.10 (3.42)
CIBIC-Plus	AG	N	3.07 (1.03) ^a^	4.08 (0.81) ^a^	N	N
	DG	N	3.69 (0.95)	4.92 (0.77)	N	N
ADCS-ADL23	AG	49.44 (11.62)	47.85 (11.22)	48.18 (11.32)	1.59 (6.11)	1.26 (6.54)
	DG	50.13 (13.57)	48.20 (13.16)	49.43 (13.45)	1.92 (3.03)	0.69 (3.05)
NPI	AG	9.28 (2.49)	7.25 (2.69)	8.13 (2.78)	2.02 (0.59)	1.15 (0.60)
	DG	8.97 (2.69)	7.61 (2.30)	9.31 (2.42)	1.36 (0.57)	−0.33 (0.58)

^a^indicates significance between the two study groups (*p* < 0.05)

N indicates not determined

**Fig. 2:**
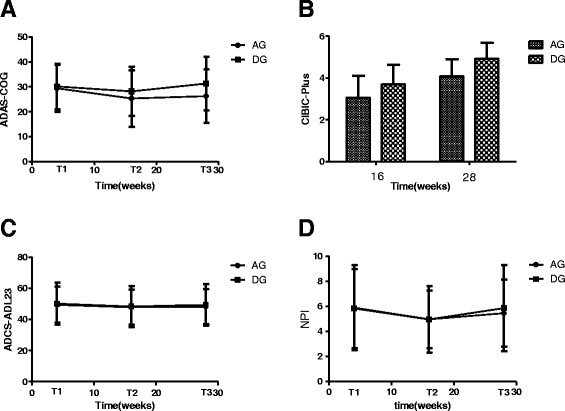
Scores of ADAS-cog, CIBIC-plus, ADCS-ADL23, NPI in AG and DG

The results of repeated measures analysis for ADAS-cog scores showed a significant time effect (*P* = 0.000) and time × group interaction(*P* = 0.000), but no group effect (*P* = 0.235) were observed. There were significant between-group differences in ADAS-cog scores at T2 and ∆ (T2-T0) (all *p* < 0.05), but no statistical differences were found at other time points (Fig. 2a).

The mean CIBIC-Plus values were 3.07 (1.03) and 4.08 (0.81) for the AG group at T1 and T2, which were much lower than that for the DG group. The CIBIC-Plus scores showed obvious decreases for AG group when compared with DG group, and significantly statistical differences were observed between the two groups (*P*<0.05) (Fig. 2b).

There were a time effect (*P* = 0.000) were found for the ADCS-ADL_23_ scores analyzed using repeated measures approach, but no treatment × time interaction (*P* = 0.421) and group effect (*P* = 0.758) were detected. No between-group differences were found in ADCS-ADL_23_ scores at any time points, and the change scores didn’t have statistical significance between the two groups (Fig. 2c).

There was a time effect (*P*=0.000), but no treatment × time interaction(P=0.05) and treatment effect(*P*=0.402) were found in the NPI scores determined with the repeated measures approach. There were not any significant between-group differences in NPI scores at any time points, and the change scores also didn’t have statistical significance between the two groups(Fig. 2d).

### Safety and tolerability outcomes

During acupuncture treatment, 4 patients(9.3%) experienced punctate hemorrhage after the needles were taken out (we stopped the bleeding by pressing for 5–10 s with the help of sterile dry cotton), and 1patient(2.3%) had bruising. No serious adverse events were reported and no patients withdrew from the trial. During donepezil treatment, 7 patients(15.9%) complained adverse events, including dizziness, nausea, loss of appetite, diarrhea, constipation, fatigue and agitation. In the 7patients, 4 cases(9.09%)withdrew from the trial and the others that had mild reaction continued to receive treatment until the end of follow up.

### Unexpected finding

In the AG group, 5 cases of insomnia, 4 cases of constipation, 6 elderly male cases with benign prostate hyperplasia and 2 cases of knee arthritis reported that their symptoms were obviously improved. However, patients in the DG group did not demonstrate equivalent improvements.

## Discussion

This exploratory, 28-week, controlled study was designed to examine the efficacy of acupuncture in the patients with mild-to-moderate AD compared to donepezil. The results indicated that acupuncture could improve cognitive function, global clinical status according to the scores of ADAS-cog, CIBIC-Plus. But the effect of acupuncture to improve activities of daily living and behavioral symptoms seemed to be limited based on the scores of ADCS-ADL_23_ and NPI.

In spite of long history and public acceptance of acupuncture, an unequivocal scientific explanation regarding to the physiological mechanism of acupuncture has not been obtained and awaits further investigation. In recent years, many research works have been done in order to offer probable evidence and promote the therapeutic use of acupuncture. For example, some studies have proven that acupuncture could enhance functional connectivity between the hippocampal regions [20],activate certain cognitive-related regions [21] and modulate default mode network activity [22] in AD patients using neuroimaging techniques. After stimulating HT7 by laseracupuncture, acetylcholinesterase activity was significantly suppressed in the hippocampus of memory-impaired rats [23]. Electro-acupuncture at DU20 could markedly ameliorate cognitive impairments, reduce aberrant overexpression of β-amyloid_1–42_, and inhibit neuronal apoptosis, significantly enhance expression levels of mature brain-derived neurotrophic factor (BDNF) and pro-BDNF in APP/PS1 mice [24]. In addition, some experiments have shown that acupuncture could improve the learning and memory abilities of senescence-accelerated mouse 8 by inducing cell proliferation in different brain regions [25], up-regulating triose phosphate isomerase activity in hippocampal tissues [26], modulating synapse functions relevant to cytoskeleton and inducing neurotransmitters secretion [27].

Furthermore, nonspecific physiological effects of acupuncture had also been reported, including neuro-physiological and neuro-chemical responses [28, 29]. In this study, we did not use sham acupuncture to exclude possible placebo effects, because it is not usually appropriate to use sham acupuncture in a pragmatic trial due to a likely detrimental effect on the trial’s ecological validity [30]. For this reason, donepezil was used as control medicine and a direct head-to-head comparison between acupuncture and donepezil was established in this study to confirm the efficacy of acupuncture in AD patients. In china, some similar studies have been done and the results have proven that acupuncture treatment was similarly effective as medication and side effects of acupuncture were far fewer than medicine. However, the results of these studies were not fully consistent and the design of the clinical trials is not very rigorous. The results of this study were substantially consistent with the published literatures [19].In this study, only minor adverse effects were observed and no patients withdrew due to side effects. However, our results could not provide evidence to prove that acupuncture therapy is better in safety than donepezil due to the small sample size. High dropout rate due to adverse effects have been reported in clinical trials with donepezil. For example, the dropout rate was 9.3% in a 15-week, double-blind, placebo-controlled study on donepezil [31]. In another open-label, multicenter, randomized trial of donepezil, the withdraw rate owing to the same reason was as high as 17% [32].In spite of minor adverse effects and low dropout rate, acupuncture could also provide some benefits for other diseases based on the theory of syndrome differentiation and treatment of TCM.

We must also note several potential limitations in this study. Firstly, the small sample size leads to underpowered results. Secondly, participants who glad to take part in this study seemed to have a positive attitude toward treatment (including acupuncture and medicine). It was possible that patients with high treatment expectations will introduce positive bias into the results of the study. At last, it is uncertain whether the findings in Tianjin city can be extrapolated to other districts.

## Conclusion

The main finding in this study was that acupuncture treatment was efficacious and well tolerated in improving cognitive function and global clinical status. Acupuncture offers a promising and effective treatment option for AD to physicians, with few adverse effects or contraindications.

## Abbreviations

AD: Alzheimer’s disease
ADAS-ADL_23_: 23-Item Alzheimer’s disease Cooperative Study Activities of Daily Living Scales
ADAS-cog: Alzheimer’s disease Assessment Scale-Cognitive
ADL: Activity of Daily Living scale
AEs: Adverse events
AG: Acupuncture group
CIBIC-Plus: Clinician’s Interview-Based Impression of Change-Plus
DG: Donepezil hydrochloride group
HAMD: 17-item Hamilton depression scale
HIS: Hanchinski ischemia scale
MMSE: Mini-mental State Examination
NPI: Neuropsychiatric Index
SAEs: Serious adverse events
T0: 4-week baseline
T1: 12-week treatment phase
T2: 12-week follow-up period
TCM: Traditional Chinese medicine
USA: united states of American
WHO: World Health Organization
